# Prospective Study of Rape Perpetration by Young South African Men: Incidence & Risk Factors

**DOI:** 10.1371/journal.pone.0038210

**Published:** 2012-05-31

**Authors:** Rachel Jewkes, Mzikazi Nduna, Nwabisa Jama Shai, Kristin Dunkle

**Affiliations:** 1 Gender & Health Research Unit, Medical Research Council, Pretoria, South Africa; 2 School of Public Health, University of the Witwatersrand, Johannesburg, South Africa; 3 Department of Psychology, University of the Witwatersrand, Johannesburg, South Africa; 4 Rollins School of Public Health, Emory University, Atlanta, Georgia, United States of America; Vanderbilt University, United States of America

## Abstract

**Background:**

There has been very little prospective research on rape perpetration among men. This paper describes the incidence and risk factors for new rape and attempted rape events among young South African men in an HIV prevention trial.

**Methods:**

We followed 1,147 men aged 15–26 years who enrolled into a cluster randomised controlled trial to evaluate the HIV prevention behavioural intervention Stepping Stones. Incidence rate ratios for factors associated with incident rape were derived from Poisson models.

**Results:**

The young men reported 217 incident rapes (completed or attempted) of a girl or woman over 1,914 person years of follow up, yielding a rape incidence of 11.2 per 100 person years. Overall 24.9% of men had previously raped at baseline, and 18.9% did so during the follow up. Among the latter, 61.3% raped for the first time, and 38.7% re-offended. Multivariable Poisson modelling showed a higher incidence of rape perpetration among men who had ever used drugs (IRR 1.86 95%CI 1.39, 2.49), had eight or more lifetime partners (IRR 1.48 95% CI 1.09, 2.01), had been physically violent toward a female partner (IRR 1.50 95%CI 1.11, 2.03) and had disclosed rape perpetration at baseline (IRR 1.45 95%CI 1.07, 1.97). A lower incidence was found among those with greater resistance to peer pressure (IRR 0.85 95%CI 0.74, 0.97).

**Conclusions:**

The findings highlight the importance of male gender socialisation and addressing delinquent youth sub-cultures in rape prevention. Prevention requires change in hegemonic masculinity, with its emphasis on gender hierarchy, exaggerated performance of heterosexuality and control of women. Interventions are needed to address male socialisation with delinquent peers, by reducing exposure to childhood trauma and strengthening opportunities for gainful employment (in work or recreation).

## Introduction

Understanding drivers of rape perpetration is critical for effective rape prevention. Research based on large samples of men from South Africa points to the central importance of gender socialisation in rape perpetration and support femininist analyses of rape [1], [2], [3], [4], [5]. Men who rape are shown as engaging in a pattern of behaviour that exaggerates heterosexual performance and emphasises control of women, including through the use of physical violence [6], [7], [8]. Men who rape have been shown to have large numbers of sexual partners, engage in transactional sex (a practice also understood as part of a pattern of control of women) [7], [9], [10], [11], [12], and use physical violence against partners (c.f. [10], [13]. South African men who rape commonly describe their rape as stemming from ideas of sexual entitlement [13], and have a pattern of ideation that justifies the rape to themselves. They hold more gender inequitable views, adversarial and hostile ideas about women and were more likely to subscribe to rape myths [2], [10], [11], [13], [14], [15].

Research has shown the context in which rape occurs to be important. Men who have raped are much more likely to have engaged with delinquent or criminal peers, often as a member of a gang, and to have also used drugs, or participated in a range of other criminal and violent behaviour [4], [13]. Thus they have commonly been part of a sub-culture in which raping is viewed as legitimate, most notably through performance in gang rapes. In South Africa gang rape is particularly common, with one study finding 20% of men interviewed had participated in gang rape either sexually (9%) or in another way (witnessing, supporting). This high prevalence, and accompanying qualitative narratives, suggests that among South African men of a particular age, gang rape perpetration is widely viewed as something ‘boys do’ [13].

For many men the origins of violence towards women have been shown to lie in childhood, with men who later rape reporting much greater exposure to abuse and adversity in childhood [10], [11], [13], [14], [16]. A psychoanalytic explanation for this is that trauma in childhood may result in disordered attachment, with affected men unable to form stable attachments to women partners, lacking empathy, having mood disturbances and low self-esteem [17]. Whilst sexual abuse in childhood is emphasised in this pattern, physical abuse is also important, although some research suggests that its impact on later perpetration of sexual coercion is mediated by delinquent activities in adolescence [18]. Sexually violent men, especially multiple offenders, are more likely to lack empathy or have remorse for their victims and to blame their victims for the rape [15], [16], [19], [20]. They are more likely to perceive they have not gotten what they deserve in life and that their peers have done better. They externalise blame, whilst acknowledging that they deliberately tested boundaries [13]. In this way they lack the internal mechanisms for controlling their own behaviour.

Most of the existing evidence on risk factors for rape perpetration arises from North America, and although there have been large studies from South Africa [10], [13], the global evidence base for understanding risk and prevention remains limited. Almost all existing research is cross-sectional (with some notable exceptions [e.g. [21]]), thus the temporal relationship of the outcome and some independent variables has often been uncertain [13], [22]. In order to develop evidence-based rape prevention strategies more evidence is needed.

Data from the Stepping Stones HIV prevention trial offers an opportunity to understand rape perpetration through longitudinal research as a large group of men were enrolled and followed up over two years. Participants were asked about rape perpetration at baseline and then annually at 12 and 24 months follow-up. These data provide an opportunity to investigate rape incidence and re-perpetration prospectively and assess risk factors for rape perpetration.

## Methods

Ethics approval was given by the University of Pretoria Faculty of Health Sciences Ethics Committee. Between 2002–2003 we enrolled youth aged 15–26 years into a cluster randomised controlled trial to evaluate the HIV prevention behavioural intervention Stepping Stones [23]. From the original 1370 enrolled men, we excluded those lost to follow up at both 12 and 24 months (n = 220), and three with missing data. We present here data from 1147 men.

The trial had two arms. One received Stepping Stones, a 50 hour participatory intervention on sexual and reproductive health and HIV, delivered over 6–8 weeks. The control intervention was 3 hours on safer sex and HIV, delivered on one occasion. In all other respects the participants in the two arms were treated no differently. They were volunteers recruited from schools in 70 locations (clusters) in the Eastern Cape province of South Africa. The clusters were divided into seven geographically-defined strata and equal numbers of clusters in each strata were randomised between the two study arms. In each cluster, 15–25 youth of each sex were enrolled (women are not discussed here). Baseline, 12 and 24 month assessments included a face-to-face questionnaire described below. All interviews were conducted by age and sex matched interviewers and most of the baseline interviews were undertaken by two men. A small group of additional interviewers were hired for the 12 and 24 month interviews, but there was considerable continuity in the team. The interviewers were very highly trained and closely supervised to try to reduce response bias.

The cohort was maintained using details collected at enrolment, with follow-up conducted nationwide as necessary to trace youth who had moved. Further information on all assessments, study recruitment, access and ethical issues, is presented elsewhere [24], [25]. All participants signed informed consent and were assured confidentiality. All were offered psychological support from a study nurse should this be required for any issue or problem via a 24 hour cell phone line.

### Questionnaire

The main outcome for this paper was perpetration of rape (completed or attempted) of a current or ex-girlfriend or any other women or girl during the follow up period. We measured rape or attempted rape against a female partner using questions about physically forced sex, sex when a woman did not want to but was afraid, and forced oral and anal sex that were modified to capture perpetration from the World Health Organisation’s instrument [26]. To measure rape of a non-partner or gang rape we asked five questions: about sex with a woman too drunk to consent or stop it, gang (or multiple perpetrator) rape, having ‘made’ a woman have sex when she didn’t want to and attempting to do this but not completing the sex act. A man was said to have raped if he responded that he had done any of these. Baseline questions covered lifetime and past year perpetration; follow-up questions covered time since previous interview. We developed these questions for this study and extensively validated them prior to use.

### Other Questionnaire Items

We asked about age, years of completed schooling, having ever earned money and having had a girlfriend. Socio-economic status was assessed using a scale derived for the study encompassing household goods ownership, food scarcity and perceived difficulty accessing money for a medical emergency (R100, about $12). We asked about the level of schooling obtained by both parents, and about orphaning. We asked whether he was active in church, how many clubs he belonged to and measured community cohesion in a five item scale developed for the study. A typical question is “In this area do most people generally trust each other in matters of lending and borrowing?” (Cronbach’s alpha 0.65).

Adverse childhood experiences were measured on a modified version of the short form of the Childhood Trauma Questionnaire [27]. It covered five dimensions of trauma: emotional neglect, emotional abuse, physical neglect/hardship, physical abuse and sexual abuse (Cronbach’s alpha 0.72).

We measured perceived susceptibility to peer pressure to have sex on a scale of three questions with 4 point response options. A typical statement was “I have to have sex because all my friends are doing it” (Cronbach’s alpha 0.72). We measured alcohol use using the AUDIT scale which has been extensively validated in developing countries. It has 12 items and asks about the frequency, quantity and consequences of drinking. We used a cut point of 8 for ‘problem drinking’. We measured drug use by asking about ever use of dagga (marijuana), benzene, mandrax, injected drugs or other drugs and dichotomised respondents as those who had ever used drugs versus those who had not used drugs. We asked about ever gang membership.

To measure lifetime number of partners we asked separately about the number of main partners, makhwapheni and once-off partners. *Makhwapheni* (pl.) is a term in local idiom and described a partner category that is definitionally concurrent for one or both of the partners involved. Number of partners was inquired for each category in the last year and lifetime. Transactional sex was defined as occurring where a man a sexual relationship that he believed to be motivated by his providing or promising the woman or girl food, cosmetics, clothes, transportation, items for children or family, school fees, somewhere to sleep, alcohol or a “fun night out”, or cash [1], [28].

A gender attitudes and relationship control scale (13-items, Cronbach’s alpha = 0.69) included questions on men’s controlling practices within a relationship and attitudes towards gender relations. The modified WHO violence against women instrument was used to measure physical partner violence perpetration (IPV) (5-items) ever [26].

We did not include variables for possible time-varying covariates during follow up because we could only do this for the one year data point and for men not reporting rape during that first year. This was too limiting to be useful in the analysis.

**Table 1:** Comparison of the sample followed up and lost to follow up.

	Followed up	Lost to follow up	p value
	n = 1147	n = 220	
	%	%	
Age (years)	19.11	19.31	0.12
Education: to grade 10	87.9 (1008)	84.1 (185)	0.18
Socio-economic status (Mean)	0.021	−0.126	0.23
Ever had a girlfriend	98.5 (1130)	93.2 (204)	<0.0001
Childhood trauma score	−0.011	0.057	0.32
Raped or attempted rape	24.9 (285)	19.1(42)	0.08
Past year physical IPV (N = 1320 partnered men)	22.2 (248)	17.3 (36)	0.12
Intervention : Stepping Stones	51.4 (590)	46.8 (103)	0.19

### Data Analysis

Analyses were conducted in Stata 10.0. All procedures took into account the study design, viewing the dataset as a cohort with a stratified, two stage structure with participants clustered within villages. For each participant we calculated the person years of exposure as the time from baseline to the last report of not raping result if the person never reported rape, or as the total time between measures of not-raping as well as half the time between the last interview where rape was not reported and first time it was reported.

The social, demographic and relationship characteristics, and violence exposures were summarised as percentages (or means) with 95% confidence limits, using standard methods for estimating confidence intervals from complex multistage sample surveys (Taylor linearization). Pearson’s chi was used to test associations between categorical variables.

To account for clustering of men within villages, a random effects Poisson model was fitted. The model included variables for age, the study treatment arm, stratum, and person years of exposure. All of the variables presented in the table showing socio-demographic and behavioural characteristics at baseline of men who did and did not rape over 2 years were candidate variables for the model. We entered the variables in groups and used backwards elimination to retain those initially with a P<0.2. Subsequently we eliminated variables with p>0.05. We tested all retained variables in the model for interactions and found none. We tested goodness of fit using the Poisson goodness of fit test. We confirmed the findings of associations for each outcome variable, by modelling survival time under observation using a Weibull model, with the same set of other variables included.

Following Greenland [29], the population attributable fractions for partner violence and low levels of relationship power equity were calculated using the incidence rates from the adjusted model and the formula PAF =  ((IR-1)/IR) x Pe where Pe was the proportion of the cases that had the exposure. Confidence intervals were calculated using the same formula but with the upper and lower confidence limits of the IRR.

## Results

Of the original set of men, 220 (16.1%) were lost to follow up. Those lost to follow up were significantly less likely to have had a girlfriend but otherwise did not differ on social and demographic characteristics or IPV or rape perpetration from those followed up (Table 1).

The mean age of the men retained was 19.1 years. They were mostly still in school (97.3%) and none had ever married. Their homes were poor and only 19.5% said people at home never went hungry. The prevalence of perpetration of rape or attempted rape at baseline was 24.9%, whilst 18.9% (217/1147 men) raped or attempted to rape during the follow up period. The 217 incident rapes occurred over 1,914 person years of follow up, for a rape incidence of 11.2 per 100 person years. Among the group reporting new rape perpetration during follow-up, 61.3% of men raped for the first time and 38.7% were re-offences. In all 6.9% of men reported rape of an intimate partner during the follow up period. 17.7% of men reported rape or attempted rape of a woman who was not a partner, including multiple perpetrator rape (2.9%) and rape of a woman who was drunk or unconscious (1%). Only 3.4% of men disclosed having completed a rape of a non-partner during the follow up period.

Those who raped during follow up differed from those who did not on a number of baseline characteristics. Although of the same age and socio-economic status, they had a higher level of school attainment and all had a girlfriend. They had a higher level of exposure to adversity and abuse in childhood. They were more susceptible to peer pressure, drank more heavily and were more likely to have used drugs. They had had more lifetime sexual partners, but were less likely to have been told they had made one pregnant. They were more likely to have had transactional sex, to have disclosed rape at baseline and to have been physically violent towards a woman (Table 2).

**Table 2:** Socio-demographic and behavioural characteristics at baseline of men who did and did not rape over 2 years.

	Rape during follow up			No rape during follow up			
	n = 217			n = 930			
	%/mean	95%CI		%/mean	95%CI		p value
**Social characteristics**							
Age (years)	19.1	18.9	19.4	19.1	18.9	19.3	0.899
Education: over grade 10	17.1 (37)	10	24.1	11.0 (102)	7.7	14.2	0.009
Ever earned money	58.3 (126)	50.00	66.70	54.0 (502)	49.9	58.1	0.29
Ever had a girlfriend	100 (217)			98.2 (913)	97.3	99	0.04
**Home background**							
Socio-economic status(mean)	−0.03	−0.27	0.22	0.03	−0.11	0.18	0.649
Childhood trauma score	0.10	−0.01	0.22	−0.04	−0.11	0.04	0.04
Mother completed school	72.2 (156)	65.2	79.2	65.7 (611)	61.8	69.6	0.06
Orphaning: none	65 (141)			66 (614)			0.91
father	25.8 (56)			25.8 (240)			
mother	6 (13)			5.7 (53)			
both	3.2 (30)			2.5 (23)			
Sexually coerced by a woman	12.4 (27)	8.2	16.7	9.0 (84)	7.1	11	0.06
**Social capital**							
Community cohesion score	0.00	−0.14	0.15	−0.05	−0.11	0.02	0.57
Active in church	43.3 (94)	36.7	50	49.4 (458)	45.5	53.2	0.073
Involvement in 1+ clubs	74.7 (162)	66.5	82.8	75.8 (705)	71.4	80.2	0.78
**Social and peer practices**							
Peer pressure resistance	−0.11	−0.25	0.03	0.05	−0.04	0.15	0.038
Problem drinking	34.6 (75)	28	41.1	23.9 (222)	20.1	27.7	0.004
Gang membership	9.2 (20)	4.4	14	5.7 (53)	4.2	7.2	0.07
Ever drug use	52.1 (113)	44.4	59.7	35.5 (330)	31.4	39.6	0.0001
**Attitudes towards and practices of gender relations**							
Gender attitudes and relationship control score	−0.02	−0.16	0.12	0.03	−0.08	0.13	0.51
Any transactional sex	37.6 (79)	29.7	45.5	27.9 (245)	23.9	31.9	0.006
8+ lifetime partners	38.1 (80)	31.5	44.7	26.0 (227)	22.5	29.5	0.001
Raped or attempted rape	38.7 (84)	31.7	45.7	21.6 (201)	18.2	25.0	<0.0001
Ever physical IPV	39.3 (84)	31.8	46.7	26.0 (232)	22.8	29.2	0.0003
**Intervention : Stepping Stones**	49.8 (108)	1.35	1.64	51.8 (482)	1.39	1.65	0.62

The Poisson model shows that a significantly higher incidence of rape perpetration during follow up was found among men had ever used drugs, had eight or more lifetime partners, had been physically violent towards a woman partner and had disclosed rape at baseline (Table 3). A lower incidence was found among those with greater resistance to peer pressure.

**Table 3:** Multivariable Poisson model of relative incidence of rape.

	Any rape during follow up: adjusted for age, education, treatment, stratum and person years of follow up (exposure)							
	IRR	95% CI		p value	% cases withthe exposure	PAF	95%CI	
Ever drug use	1.86	1.39	2.49	<0.0001	52.1	24.1	14.6	31.3
8+ lifetime partners	1.48	1.09	2.01	0.012	38.5	12.5	3.2	19.4
Ever physical IPV	1.50	1.11	2.03	0.009	39.3	13.1	3.9	19.9
Raped or attempted rape at baseline	1.45	1.07	1.97	0.016	38.6	12.0	2.5	19.0
Peer pressure resistance	0.85	0.74	0.97	0.016				

The population attributable fractions (table 3) show that 24% of incident rapes were associated with and potentially preventable by reduction in recent drug use. Similarly, 13% of new rapes were attributable to previously perpetrated physical partner violence, and 12% were attributable to previous rapists re-offending. 12% of rapes were attributable to high numbers of sexual partners.

## Discussion

Rape perpetration is highly prevalent in South Africa [13]. One in five men young South African men enrolled in this study raped or attempted to rape over the two year period of follow up, with a total rape incidence of 11.2 per 100 person years. More than a third of these perpetrators had previously raped, which supports observations from North American and other South African studies that men who rape commonly do so on multiple occasions (although they less frequently are arrested on multiple occasions) [30] [13], [31]. After adjusting for other factors, the incidence of rape was 45% higher among those who had previously raped. The Population Attributable Fraction indicates that 12% of rape in this sample was due to re-perpetration. This highlights the importance of early interventions for primary prevention of rape.

The risk factors leading to a higher incidence of rape strongly point to the importance of understanding culture, sub-cultures, gender socialisation, and context in rape perpetration. Men who raped were much more susceptible to peer pressure, something that is particularly important given the peer context within which much rape, especially South Africa’s highly prevalent gang rape, is perpetrated [10], [32]. Men’s susceptibility to peer pressure when combined with association with delinquent peers, results in exposure to misogynous and antisocial male adolescent sub-culture. This has been described in qualitative research from the region and often includes acts of theft and interpersonal violence between men [33].

The variable that had the highest population attributable fraction was drug use. The analysis shows that a quarter (24%) of all rape could potentially be eliminated if the drug use variable was not present among the men. Although questions were asked about use of a range of drugs, only dagga (cannabis) and sniffing benzene were common. Little has been published on the relationship between drug use and rape [34], but cannabis use has been found to be associated with sexual coercion in male college students in the United States [21]. They argued that it was very likely that drug use and sexual aggression co-occurred in men who lead a generally deviant lifestyle and that there was no evidence that the effect was pharmacological [21]. In this dataset we concur, especially given the very high population attributable fraction, which otherwise would be very unlikely with a weak measure of drug use. Thus this variable is most likely to be an indicator variable for the context of use: an indicator of male socialisation in a sub-culture that is unperturbed by the illegality and social condemnation of drug use in this area. In the area of this study, drugs are used by many bored, delinquent young men who can be seen in townships and villages hanging out in groups on the streets or near the village stores, in the words of one “watching life happen” [33] [35]. This is a critical context in which rape is perpetrated. Thus we suggest that the drug use variable conveys a greater propensity to rape because it is an indicator of context, rather than of physiological impact of drugs.

Social learning around the acceptability of sexual violence within sub-cultural contexts is important in rape perpetration. This partly explains the strong connection between gang membership, or delinquent peer association, and rape perpetration that has been described in diverse settings (e.g [4], [10], [13], [36]. Also the observation that across settings, a high proportion of adolescent rapes have multiple perpetrators [10], [13], [36]. Indeed incarcerated American adolescents who have raped are nearly four times as likely as non-perpetrators to know a perpetrator of sexual assault [37]. Whilst the context of association with delinquent and anti-social peers provides a sub-cultural context in which aggressive and criminal conduct is modelled, interventions to reduce sexual violence are essential in programmes aimed at this group of youth [13] [11], [18].

The importance of emphasised heterosexuality and legitimated violence against women in rape perpetration are seen in the measure of physical intimate partner violence perpetration. Men who had been physically violent towards female partners at baseline were 50% more likely to rape and the Population Attributable Fraction indicates that 13% of rape in this sample could have been prevented if the use of physical IPV were prevented. This perhaps lower than might have been expected and coupled with the unexpected finding that there was not significant interaction between baseline rape perpetration and physical IPV perpetration, it suggests that there is less overlap between the practices of physical IPV and rape than might have been assumed.

Emphasised heterosexuality has been described as a central feature of a masculinity that is hegemonic among African men predicated on the idea of women’s subordination to men and subject to their control [6], [38]. This masculinity encompasses conspicuous displays of heterosexuality, ideas of male sexual entitlement and may legitimate the use of violence against women to achieve and emphasise the gender hierarchy [6]. The connection between dominant gender socialisation of young men and raping indicates that rape cannot just be explained in terms of delinquent sub-culture, although this is important, but also points to ways in which some aspects of the mainstream culture among adolescent boys enhances the propensity to rape. The Stepping Stones intervention which was being evaluated in the trial sought to be gender transformative. There was arguably some evidence that it did impact on rape perpetration at 12 months, aOR 0.71 (95%CI 0.47 to 1.06) p = 0.094, although the study lacked power to evaluate this properly and some additional caution is need as it was a secondary outcome [25]. This, however, suggests that there should be further research into the ability of gender transformative interventions to impact on rape perpetration.

Given its importance in cross-sectional research on rape [39], one variable that was surprisingly not associated with incident rape in this study was exposure to childhood trauma. This was not retained in the model after the inclusion of terms for baseline disclosure of rape, physical IPV and drug use, although the incidence was elevated before adjustment for these. Since these three practices are all behaviours that develop subsequent to childhood trauma exposure this finding should not be interpreted as refuting hypotheses of the importance of exposure to trauma in childhood in practices of rape perpetration.

The key strength and contribution of this study lies in the fact that the data was collected prospectively with a large sample of men. While the study participants were volunteers and so may have differed from men in the general population in subtle and unknown ways, we have no reason to believe this would have made much difference to the estimates of association. The data may have been vulnerable to reporting bias. The proportion of men disclosing any rape or attempted rape during follow up was high, and commensurate with the very high prevalence of rape found at baseline, however the proportion disclosing completed rape during follow up (as opposed to attempts) was much lower than expected from the high prevalence and evidence of past year prevalence in other South African male populations (about 5%) [13] [40]. This may have been influenced by the face to face interview context of data collection, fears of legal sanction or disapproval, and a perception among men in the intervention arm that they should have behaved better. The impact of reporting bias will have been reduced by the decision to present the analysis here for rape and attempted rape and not just focus on men who disclosed having completed rapes during the follow up period. There was some loss to follow up as this was a longitudinal study. There was very little difference between those retained and lost to follow up on social and demographic characteristics and no evidence of differential loss by baseline reports of violence against women. Thus we have no reason to assume that those lost to follow up would necessarily differ from those retained in the study.

Ethical aspects of this research are of interest because it was a cohort and so the responses could potentially have been linked back to individuals. It was essential that confidentiality was assured and data kept securely and at a different site from the computer with the database of contact details. The latter was destroyed as soon as it was no longer needed. Questionnaires did not collect information that could have enabled an admission of rape perpetration to be linked by a third party to a specific act of rape and so disclosure did not really pose a risk to study participants. Interviewers were trained in non-collusive interviewing so that they did not appear to endorse or minimise rape during the interview questioning.

### Conclusion

This paper provides valuable evidence to support arguments that rape prevention must focus on changing constructions of masculinity prevailing across a mainstream culture in a society like South Africa, as well as addressing delinquent sub-cultures. To address the former, interventions are needed to promote gender equity and change hegemonic masculinity, with its emphasis on heterosexual performance and control of women. To address the latter, it is critically important to address the antecedents of youth gangs, particularly exposure to trauma in childhood, that has been well established as leading many young men to become involved in delinquent peer activities. It is also important for rape prevention to promote policies and programmes in the educational and other sectors to get young men off the streets and into work, or if at school, into gainful activity recreational activities.
